# The effects of intragastric infusion of umami solutions on amygdalar and lateral hypothalamic neurons in rats

**DOI:** 10.14814/phy2.12545

**Published:** 2015-10-05

**Authors:** Munkhzul Davaasuren, Jumpei Matsumoto, Choijiljav Chinzorig, Tomoya Nakamura, Yusaku Takamura, Enrico Patrono, Takashi Kondoh, Taketoshi Ono, Hisao Nishijo

**Affiliations:** 1 System Emotional Science, Graduate School of Medicine and Pharmaceutical Sciences, University of ToyamaToyama, Japan; 2 Institute for Innovation, Ajinomoto Co., Inc.Kawasaki, Japan

**Keywords:** Amygdala, glutamate, lateral hypothalamus, postingestive effects, vagus nerve

## Abstract

Previous behavioral studies have suggested that l-glutamate, an umami substance, is detected in the gut, and that this information regarding glutamate is conveyed from the gut to the amygdala and the lateral hypothalamus (LH) through the vagus nerve to establish glutamate preference. In this study, we investigated the roles of the amygdala and LH in the information processing of gut glutamate. We recorded the activity of amygdalar and LH neurons during the intragastric administration of five test solutions (monosodium l-glutamate [MSG, 60 mmol/L]; inosine monophosphate [IMP, 60 mmol/L]; a mixture of MSG and IMP; NaCl [60 mmol/L]; or physiological saline) in intact and subdiaphragmatic vagotomized awake rats. In intact rats, 349 and 189 neurons were recorded from the amygdala and LH, respectively, while in vagotomized rats, 104 and 90 neurons were recorded from the amygdala and LH, respectively. In intact rats, similar percentages of neurons (30–60%) in the amygdala and LH responded to the intragastric infusion of the solutions. Vagotomy significantly altered responses to the MSG and NaCl solutions. In particular, vagotomy suppressed the inhibitory responses to the NaCl solution. Furthermore, vagotomy increased the response similarity between the MSG and NaCl solutions, suggesting that vagotomy impaired the coding of the postingestive consequences of the MSG solution in the amygdala and LH, which are unique for glutamate. The present results provide the first neurophysiological evidence that amygdalar and LH neurons process glutamate signals from the gut.

## Introduction

Ingested nutrients are sensed in the gut as well as the oral cavity. Nutrient information from the gut is conveyed to the brain and can influence feeding behavior, food preference, and emotional states (Berthoud 2008; Mayer 2011; Damasio and Carvalho 2013). Consistent with this idea, in the conditioned flavor preference paradigm, animals develop a preference for a flavored solution if the flavored solution is paired with an intragastric infusion of nutrients, suggesting that intraluminal nutrients are rewarding (Sclafani 2001). l-glutamate is an umami substance that rats prefer by oral intake (Kondoh et al. 2000; Ho et al. 2011). Furthermore, ingestion of a flavored solution paired with intragastric administration of monosodium l-glutamate (MSG) can induce a preference for the flavored solution in rats (Uematsu et al. 2009, 2010). These findings suggest that visceral information from the gut plays an important role in motivated feeding behaviors.

The amygdala and the lateral hypothalamus (LH) are important forebrain areas involved in motivated behaviors. The amygdala is critical for emotion (Nishijo et al. 1988a,b; LeDoux 1995), and amygdalar lesions alter food preferences in monkeys and rats (Isaacson 1982; Murray et al. 1996). The LH is also important for food reward and motivated feeding behavior (Harris et al. 2005; Figlewicz and Benoit 2009). Neurophysiological studies have reported that amygdalar and LH neurons differentially respond to food or cues associated with reward, and nonfood (Rolls 1978; Fukuda et al. 1986; Ono et al. 1986; Nishijo et al. 1988a,b, 1998). A recent study reported that these brain regions are also involved in conditioned flavor preference; rats with amygdalar and LH lesions failed to associate a flavor with the intragastric administration of carbohydrates (Touzani and Sclafani 2005). Neurophysiological studies have reported that intragastric infusions of MSG and inosine monophosphate (IMP), both of which are umami substances, activated vagal afferent activity (Niijima 2000; Uneyama et al. 2006; Kitamura et al. 2011). Studies using functional magnetic resonance imaging (fMRI) and c-Fos have reported that intragastric MSG infusions can activate the amygdala and LH (Tsurugizawa et al. 2008, 2011; Otsubo et al. 2011). In addition, subdiaphragmatic vagotomy suppressed both the acquisition of conditioned flavor preference and the brain activation induced by intragastric MSG infusion in rats (Tsurugizawa et al. 2009; Uematsu et al. 2010). These results suggest that nutritional glutamate information from the gut is conveyed to the amygdala and LH through the vagus nerve to induce motivated feeding behaviors.

Together, these data suggest that amygdalar and LH neurons can respond to the presence of intraluminal glutamate. It is noted that fMRI and Fos expression do not directly measure postsynaptic electrical activity; fMRI signal does not covary with neuronal firing rates (Logothetis and Pfeuffer 2004) and Fos expression could be induced without neuronal depolarization (Numan 2014). Furthermore, several drawbacks of these methods to detect postsynaptic electrical activity have been discussed (Chaudhuri 2007; Bandettini 2009). Therefore, direct neurophysiological evidence that supports this hypothesis is lacking. However, no previous studies have investigated single neuronal responses in the amygdala and LH during the intragastric glutamate infusion. Furthermore, the amygdala and LH receive visceral information not only through the vagus nerve but also through the splanchnic nerve, as well as various other humoral factors (Mayer 2011; Critchley and Harrison 2013). The specific role of the vagus nerve in processing glutamate information from the gut remains unknown. In the present study, we investigated these issues by recording amygdalar and LH neurons in awake rats during the intragastric infusion of MSG and IMP. Furthermore, we analyzed the effects of vagotomy on the neural responses to intragastric MSG infusion.

## Materials and Methods

### Subjects

Adult male Wistar rats (*N *=* *54, 230–430 g; SLC, Japan) were used for these studies. Eleven rats received a subdiaphragmatic vagotomy (SVX) during the surgery (SVX rats), while the remaining 43 rats did not receive SVX (intact [non-SVX] rats). Housing temperature was maintained at 23 ± 1°C, with a 12-h light/dark cycle (lights on at 07:00). Prior to surgery, two male rats were housed per cage; after surgery, rats were individually housed, with food and water available ad libitum. All rats were treated in strict compliance with the United States Public Health Service Policy on Human Care and Use of Laboratory Animals, the National Institutes of Health Guide for the Care and Use of Laboratory Animals, and the Guidelines for the Care and Use of Laboratory Animals at the University of Toyama. All experimental procedures were approved by our institutional committee for experimental animal ethics. Every attempt was made to minimize the number of experimental animals and their suffering.

### Surgery

Rats were anesthetized with sodium pentobarbital (40 mg/kg, i.p.). Electrode assemblies were implanted either unilaterally or bilaterally into the amygdala (2.1 mm caudal from the bregma, 4.0 mm lateral from the midline, and 7.1 mm below the brain surface) and/or the LH (2.1 mm caudal from the bregma, 2.0 mm lateral from the midline, and 8.1 mm below the brain surface), according to the atlas of Paxinos and Watson (Paxinos and Watson 2006). The recording electrode assembly comprised four tetrodes, each of which included four tungsten microwires (20 *μ*m in diameter; California Fine Wire), encased in a stainless steel cannula (30 gauge; Hakko), and a microdrive. The tip impedance was approximately 200 kΩ at 1 kHz. For intragastric cannulation, a midline incision was made in the abdominal wall. One end of a silicon tube was inserted into the gastric fundus and ligated with a silk thread. The other end of the silicon tube was passed from the abdomen under the back skin and held on the skull (Tsurugizawa et al. 2008). In SVX rats, the dorsal and ventral trunks of the vagus nerve were cut at a level just under the diaphragm (Smith and Jerome 1983), while the vagus nerve was intact (not cut) in the intact rats. The muscles and skin were subsequently sutured. After surgery, all rats were allowed to recover for 1 week.

### Experimental setup

**Figure 1:**
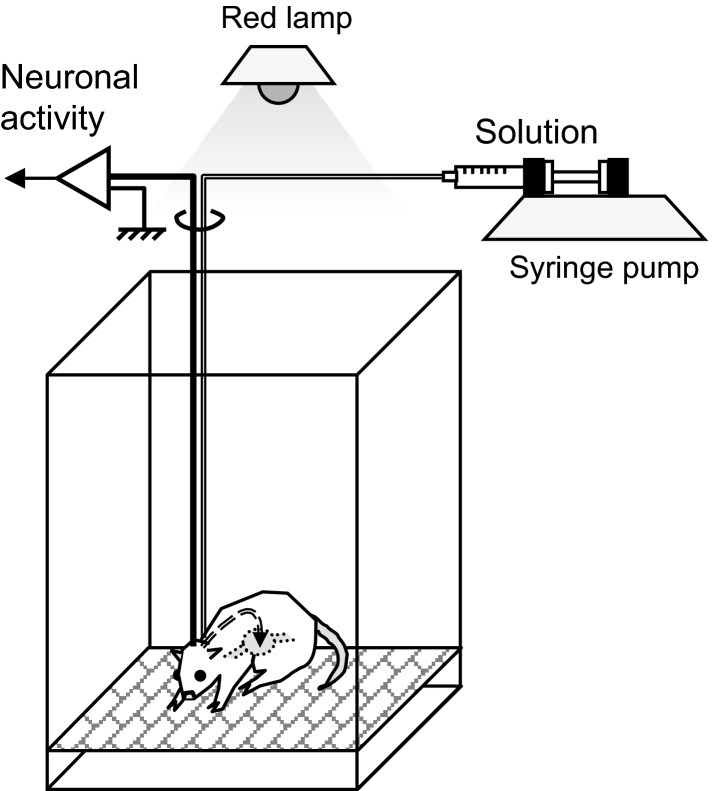
Experimental setup. These experiments were conducted in a dim room illuminated by a red lamp. Test solutions were delivered through a gastric cannula using a syringe pump. Neural activity was recorded through a cable connected to the subject rat’s head. The rat could freely move in the chamber.

The experimental setup used in this study is detailed in Figure1. Neuronal recordings were performed in a dim room illuminated by a red lamp. An acrylic chamber (30 × 40 × 40 [width × length × height] cm) was used for the recording; a stainless steel mesh floor was attached 2 cm above the acrylic floor. Various solutions were administrated into the stomach using a gastric cannula and a syringe pump (CFV-3100; Nihon Kohden, Tokyo, Japan). The amplified analog signals of neuronal activities were digitized at a 40-kHz sampling rate. Any 0.8-msec waveforms that crossed an experimenter-defined threshold were stored on a computer hard disk for offline spike sorting via OmniPlex (Plexon Inc., Dallas, TX).

### Recording procedures

One to three hours before the recording, physiological saline (1 mL) was flushed through the intragastric cannula to clean the inside of the cannula. The recording procedure (detailed below) was conducted daily during the dark phase (7:00 pm–00:00 pm) after 10–14 h of fasting. First, a cable was connected to the socket, which was connected to the electrodes on the subject rat’s head. The rat was then placed in the recording chamber and neuronal activity was checked. If stable neuronal signals were identified over a 10-min period, the recording was started. If no signal was found, the recording was not conducted on that particular day and the electrode assembly was lowered by approximately 20–100 *μ*m. After a 20-min baseline recording, 1 of 5 test solutions (MSG, 60 mmol/L; IMP, 60 mmol/L; a mixture of MSG and IMP [MSG + IMP, each 60 mmol/L]; NaCl, 60 mmol/L; or physiological saline) was delivered into the stomach via the implanted cannula for 10 min at a rate of 1 mL/min per kg body weight. It is reported that both oral intake and gastric infusion of 60 mmol/L MSG were rewarding in rats (Kondoh et al. 2000; Uematsu et al. 2009), while gastric infusion of 30–150 mmol/L MSG and 30 mmol/L IMP increased activity of vagal nerve afferents (Uneyama et al. 2006; Kitamura et al. 2011), and gastric infusion of 60 mmol/L MSG activated the amygdala and LH in an fMRI study (Tsurugizawa et al. 2008). After the 10-min solution administration, the neuronal recordings were continued for an additional 20 min. The animals stayed quiet and sometimes performed self-grooming throughout the recording. No obvious difference in behaviors between before and after the administrations was recognized. During a single daily recording, only one of the test solutions was administered. In some neurons, the sensitivity to phasic stomach distention was tested, in addition to the test solutions. During this test, physiological saline (5 mL) was administered into the stomach for 30 sec; the neural activity during 5 min before and after the administration onset was recorded.

Neuronal activity was recorded from a same electrode location for a maximum of 5 days to evaluate all the test solutions. Subsequently, the electrode assembly was lowered to the next location by at least 80–100 *μ*m to prevent recording from the same neuron.

### Data analysis

#### Spike sorting

Digitized neuronal activity was isolated into single units by waveform components according to the previous standard methods (Lewicki 1998; Harris et al. 2000; Nicolelis 2008), using the Offline Sorter program (Plexon Inc.). Briefly, each of the recorded waveforms was plotted in two- or three-dimensional feature spaces; various features of spike waveforms (waveform projection onto principal components, peak amplitudes of the waveforms, valley amplitudes of the waveforms, peak-valley amplitudes of the waveforms, etc.) can be selected as a dimension. In the feature space, waveforms with a similar shape, which come from a same neuron, are appeared in a cluster, while waveforms with different shapes, which come from different neurons, are appeared in different clusters. Thus, spikes in each cluster in the feature space were considered as a single unit if they passed the following four criteria: (1) the cluster boundaries were well separated from the other clusters; (2) waveform shapes in the cluster were consistent; (3) the waveform shapes were consistent with the action potentials; (4) an absolute refractory period of at least 1.5 msec was observed in an interspike interval histogram. The isolated single units were then transferred to the NeuroExplorer program (Nex Technology, Littleton, MA) for further analysis. Typically, 1–4 single units were isolated by offline cluster analysis from the four channels (wires) of one tetrode.

#### Neuronal responses to the test solutions

Responses to the test solutions (MSG, IMP, MSG + IMP, NaCl, or physiological saline) were analyzed in each neuron. The pre- and postadministration periods were defined as 20 min before and 30 min after the onset of the infusion, respectively. A firing rate histogram during these periods (bin width = 30 sec) was computed for each solution. The baseline firing rate of a neuron was calculated from the preadministration period. A neuron was considered to be responsive to a given solution if three successive bins in the postadministration period of the histogram deviated from the baseline firing rate by ±2 standard deviations (SD). Responsive neurons were further subgrouped into excitatory, inhibitory, and complex (showing both excitatory and inhibitory characteristics) responsive neurons, according to the direction of the deviation. For each responsive neuron, the response latency (time when three successive bins first deviated from the baseline firing rate by ±2 SD), the duration (the number of bins deviating the baseline firing rate by ±2 SD), and the magnitude (mean firing rate during the postadministration period minus the baseline firing rate) were computed.

#### Neuronal responses to stomach distention

Some neurons were tested with a 5-mL saline injection to analyze the sensitivity to phasic stomach distention. The pre- and postadministration periods for this analysis were defined as 5 min before and after the onset of the administration, respectively. A firing rate histogram around the administration of the solution during these periods (bin size = 30 sec) was computed. The baseline firing rate of a neuron was calculated from the bins within the preadministration period. A neuron was considered to be responsive to phasic stomach distention if the first bin (0–30 sec) or second bin (30–60 sec) after the administration onset in the histogram deviated from the baseline firing rate by ±2 SD.

#### Comparisons between the responses to the test solutions in intact rats

The ratios of the three types of responsive neurons (e.g., excitatory, inhibitory, and complex) and nonresponsive neurons were compared between the solutions using a *χ*^2^ test (df = 3). Subsequent multiple post hoc comparison analyses were performed with residual analysis. Each response parameter (latency, duration, and magnitude) of the excitatory and inhibitory responsive neurons was compared using a two-way analysis of variance (ANOVA) with two factors: 2 brain regions (amygdala and LH) × 5 solutions (MSG, IMP, MSG + IMP, NaCl, and physiological saline). The two-way ANOVAs were performed using types II and III sum of squares, both of which were effective to decrease confounding due to unequal sample sizes (Shaw and Mitchell-Olds 1993). Since two-way ANOVAs using both types II and III sum of squares indicated similar statistical significance, we noted only the results derived from the two-way ANOVA using type III sum of squares. Subsequent multiple post hoc comparison analyses were performed with the Turkey’s test. These statistical tests were conducted with SPSS Statistics 19 or Microsoft Excel 2010. *P*-values less than 0.05 were considered statistically significant.

#### Comparisons between the responses of intact and SVX rats

The ratios of the three types of responsive neurons (i.e., excitatory, inhibitory, and complex) and nonresponsive neurons for each solution (MSG and NaCl) were compared between the intact and SVX rats using a *χ*^2^ test (df = 3). Subsequent multiple post hoc comparison analyses were performed with residual analysis. Each response parameter (latency, duration, and magnitude) of the excitatory and inhibitory responsive neurons was also compared using a three-way ANOVA with three factors: 2 groups (intact vs. SVX rats) × 2 brain regions (amygdala vs. LH) × 2 solutions (MSG vs. NaCl). The three-way ANOVAs were performed using types II and III sum of squares. Since the three-way ANOVAs using both types II and III sum of squares indicated similar statistical significance, we noted only the results derived from the three-way ANOVA using type III sum of squares. Subsequent multiple post hoc comparisons were performed using simple main effect analyses focusing on the difference between the intact and SVX rats.

#### Comparisons between the responses to MSG and NaCl solutions

We have tested the putative same neurons with the spike waveforms that were stable across days (Jackson and Fetz 2007). To check the stability, the consistency of the averaged waveforms and the cluster location in the feature spaces over the time were visually inspected. Furthermore, the stability was quantitatively assessed by calculating Pearson’s correlation coefficient between the averaged waveforms in 2 days (Jackson and Fetz 2007). If the correlation coefficient between the two waveforms was more than 0.9, the waveforms were considered to be stable. Among all the neurons analyzed by the above methods, some neurons passed these criteria of waveform stability. In each stable neuron, the response similarity between the MSG and NaCl solutions was assessed as follows. First, the firing rate histograms before and after the infusion of each solution were smoothed by a moving average; a given averaged bin was defined as the mean of 13 bins (the given bin and the six adjoining bins for 3 min). The response similarity was defined as a Pearson’s correlation coefficient between the corresponding bins in the smoothed histograms for the MSG and NaCl solutions. The mean response similarities were compared with a two-way ANOVA with two factors: 2 groups (intact and SVX rats) × 2 brain regions (amygdala and LH).

### Histology

After neural recordings, rats were deeply anesthetized with pentobarbital sodium (50 mg/kg; i.p.), and the recording sites were marked with electrolytic lesions by passing a 20-*μ*A negative current through the recording electrodes for 30 sec. The rats were then perfused transcardially with 0.9% saline, followed by 10% buffered formalin containing 2% potassium ferricyanide. The brain was removed and fixed in formalin for at least 48 h. Serial sections (60 *μ*m) were cut on a freezing microtome and stained with cresyl violet.

## Results

### Basic response characteristics of amygdalar and LH neurons in intact rats

**Figure 2:**
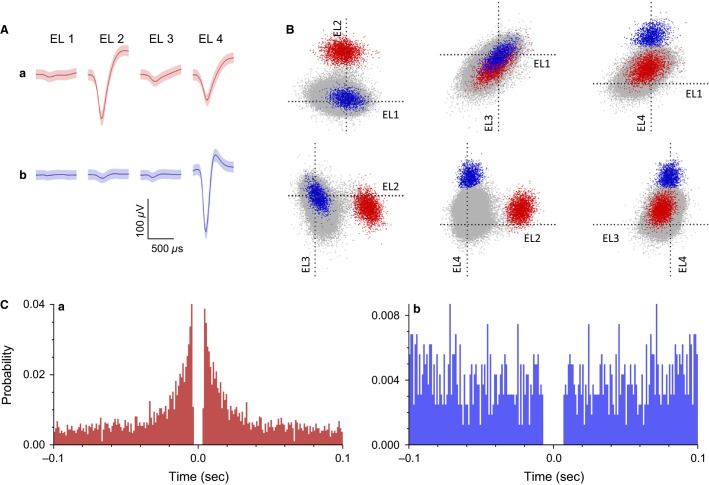
Example waveforms of two amygdalar neurons isolated by an offline cluster analysis. (A) Waveforms (mean ± SD, shaded) recorded from four electrodes (tetrode) (EL 1–4). The waveforms indicated by a and b correspond to the two neurons (a and b), respectively, identified by the offline cluster analysis in B. (B) The results of an offline cluster analysis. Each dot represents one neuronal spike. The axes represent the first principle component of each of the four electrodes. Two colored clusters (red and blue, corresponding units a and b in A, respectively) are recognized. (C) Autocorrelograms of the neurons. Bin width = 1 msec. Ordinates indicate probability, where bin counts were divided by the number of spikes in the spike train.

In intact rats, 349 and 189 neuronal activities were recorded from the amygdala and LH, respectively. Typical waveforms of two amygdalar neurons (a, b) were simultaneously recorded from four wires in the same tetrode (EL 1–4) (Fig.2A). Figure2B displays the results of spike sorting by offline cluster cutting of the neuronal activities shown in Figure2A. Each dot represents one spike, and three clusters of dots indicated by different colors were recognized. Units a, b in Figure2B correspond to the two single amygdalar neurons (a and b, respectively) shown in Figure2A. Autocorrelograms of these neurons indicate that their refractory periods were more than 3 msec, which demonstrates that these spikes were recorded from single neurons (Fig.2C).

**Figure 3:**
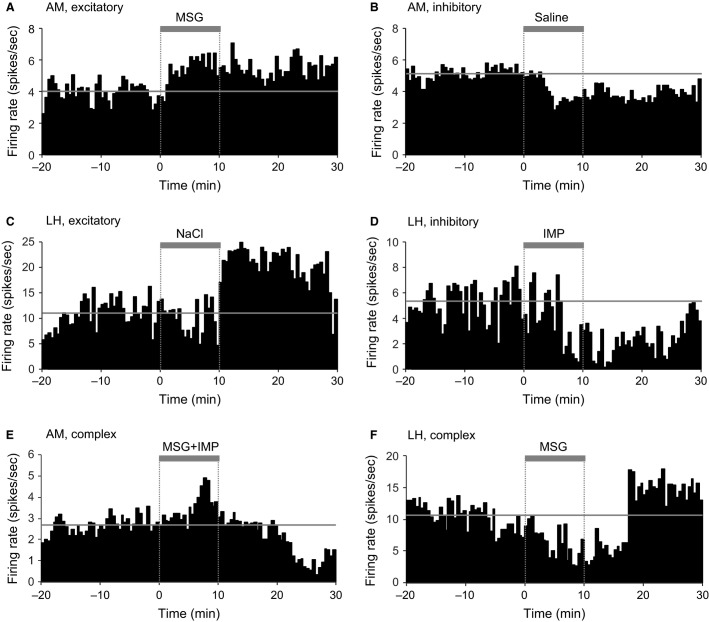
Examples of neural responses to intragastric administration of solutions in intact rats. Perievent histograms of three amygdalar (AM) neurons (A, B, E) and three LH neurons (C, D, F). Time 0 indicates the onset of solution administration. Thick gray bars on the histogram indicate solution administration. Horizontal thin gray lines indicate the mean baseline firing rates.

Figure3 presents examples of the neuronal responses from excitatory (A and C), inhibitory (B and D), and complex (E and F) responsive neurons in intact rats. Table1 summarizes the number of responsive and nonresponsive neurons to each test solution in each brain region in intact rats. Only seven neurons showed the complex responses (e.g., Fig.3E and F). Among the complex responsive neurons, four neurons showed excitatory and then inhibitory responses (AM, 3; LH: 1; Fig.3E), two neurons showed inhibitory and then excitatory responses (AM: 1; LH: 1; Fig.3F), and one neuron showed inhibitory, excitatory, and then inhibitory responses (AM: 1). There were some differences in the ratios of the responsive neurons in the amygdala and LH, and between the test solutions. First, the ratios of the responsive neurons to the test solutions were compared with the ratios of the responsive neurons to physiological saline in each brain region (Table1a). This analysis indicated that in the amygdala, the ratio of the excitatory IMP-responsive neurons was significantly greater than the ratio of saline-responsive neurons. Second, the ratio of the responsive neurons to the MSG, IMP, and MSG + IMP solutions were compared to the ratio of the responsive neurons to the NaCl solution (Table1b). This analysis indicated that in the amygdala, the ratio of the inhibitory MSG-responsive neurons was significantly smaller than the inhibitory NaCl-responsive neurons. Third, the ratios of the responsive neurons to the same solutions were compared between the amygdala and LH (Table1c). This analysis indicated that the ratio of the inhibitory IMP-responsive neurons was significantly smaller in the amygdala than in the LH. These analyses revealed significant differences in the ratios of the responsive neurons only for some solutions, suggesting that the amygdala and LH neurons showed similar responsiveness to the solutions. On the other hand, the ratios of excitatory and inhibitory neurons were different between the amygdala and LH. In the amygdala, 98, 38, and 5 neurons showed excitatory, inhibitory, and complex responses, respectively, while in the LH, 40, 38, and 2 neurons showed excitatory, inhibitory, and complex responses, respectively. Among the responsive neurons, the ratios of the excitatory neurons were significantly higher in the amygdala (69%) than in the LH (50%) (residual analysis, standard residual = 2.839). The ratios of the inhibitory neurons were higher in the LH (48%) than in the amygdala (27%) (residual analysis, standard residual = 3.055).

**Table 1:** The number of responsive neurons to each solution in intact rats

	MSG	IMP	MSG + IMP	Saline	NaCl
AM					
Excitatory	25 (22%)	25 (42%)^a^	8 (24%)	15 (23%)	24 (32%)
Inhibitory	8 (7%)^b^	3 (5%)^c^	4 (12%)	10 (15%)	13 (17%)
Complex	1 (1%)	1 (2%)	1 (3%)	1 (2%)	1 (1%)
No response	80 (70%)^b^	31 (52%)	20 (61%)	40 (61%)	38 (50%)
Total	114	60	33	66	76
LH					
Excitatory	9 (16%)	9 (26%)	5 (16%)	11 (29%)	6 (20%)
Inhibitory	6 (11%)	11 (32%)^c^	7 (22%)	9 (24%)	5 (17%)
Complex	1 (2%)	0 (0%)	1 (3%)	0 (0%)	0 (0%)
No response	39 (71%)^a^	14 (41%)	19 (59%)	18 (47%)	19 (63%)
Total	55	34	32	38	30

a, b, c Significant difference from the ratios of responsive neurons to saline (a) or NaCl (b) in the same brain region, or those to the same solution in the other brain region (c) (residual analysis, standard residual > 2.0).

AM, amygdala; LH, lateral hypothalamus; Saline, physiological saline.

Table2 summarizes the basic response characteristics of the responsive neurons. To analyze the differences in their response patterns, a two-way ANOVA (brain region × solution) was performed for each parameter of the excitatory and inhibitory responsive neurons. In regard to the latencies of the excitatory responsive neurons, there were significant main effects for brain region (amygdala: 10.2 ± 0.2 min, LH: 12.8 ± 1.3 min; *F*(1, 127) = 4.33, *P *=* *0.039) and for duration (amygdala: 9.9 ± 0.6 min, LH: 7.6 ± 0.9 min; *F*(1, 127) = 5.84, *P *=* *0.017). In the inhibitory responsive neurons, there was a main effect of magnitude (amygdala: −2.9 ± 0.7 spikes/sec; LH: −10.2 ± 2.2 spikes/sec; *F*(1, 66) = 10.05, *P *=* *0.002). These results indicate that the response patterns were different in the amygdala and in the LH. Furthermore, there was a significant main effect of solution in regard to the response duration of the inhibitory responsive neurons (*F*(4, 66) = 4.27, *P *=* *0.004). Post hoc comparisons revealed that the response durations were significantly shorter for the MSG solution (8.2 ± 1.2 min) compared to the saline solution (15.2 ± 1.7 min) (Turkey’s test, *P *=* *0.005). However, no significant interactions between brain region and solution were identified, indicating that the difference in the duration did not depend on brain regions.

**Table 2:** Basic response characteristics of the excitatory and inhibitory neurons in intact rats

	MSG	IMP	MSG + IMP	Saline	NaCl
AM					
Excitatory					
Latency (min)^c^	10.4 ± 1.6	9.9 ± 1.7	6.3 ± 1.4	12.8 ± 2.5	10.1 ± 1.4
Duration (min)^c^	9.7 ± 1.0	9.8 ± 1.4	12.9 ± 2.9	7.1 ± 1.0	10.8 ± 1.3
Magnitude (spikes/sec)	1.5 ± 0.8	0.7 ± 0.4	0.4 ± 0.2	0.8 ± 0.4	1.4 ± 0.4
Inhibitory					
Latency (min)	9.2 ± 1.9	14.2 ± 2.7	10.0 ± 4.3	13.0 ± 3.0	16.0 ± 2.3
Duration (min)	8.2 ± 1.5^a^	5.5 ± 1.3	11.9 ± 3.7	14.2 ± 2.5	9.9 ± 1.8
Magnitude (spikes/sec)^c^	−1.8 ± 1.0	−1.5 ± 1.2	−5.0 ± 2.6	−3.0 ± 1.6	−3.0 ± 1.1
LH					
Excitatory					
Latency (min)^c^	13.1 ± 2.8	10.4 ± 2.2	13.9 ± 5.0	11.5 ± 2.7	17.3 ± 3.1
Duration (min)^c^	8.1 ± 2.7	7.3 ± 1.3	4.4 ± 0.4	9.5 ± 1.7	6.7 ± 2.4
Magnitude (spikes/sec)	1.3 ± 0.9	1.6 ± 0.6	0.5 ± 0.3	0.5 ± 0.6	2.1 ± 1.0
Inhibitory					
Latency (min)	13.6 ± 3.6	11.9 ± 2.4	15.4 ± 2.0	8.5 ± 2.1	10.3 ± 4.4
Duration (min)	7.8 ± 2.0^a^	13.0 ± 2.1	8.9 ± 1.7	18.4 ± 2.1	17.3 ± 4.1
Magnitude (spikes/sec)^c^	−8.2 ± 4.4	−8.0 ± 2.8	−6.9 ± 2.4	−10.9 ± 5.2	−20.9 ± 11.2

Data are expressed as mean ± SEM.

a, b, c the value is significantly different from saline (a) or NaCl (b) in the same brain region or same solution in the other brain region (c) (Tukey’s test, *P *<* *0.05).

AM, amygdala; LH, lateral hypothalamus; Saline, physiological saline.

### Responsiveness to phasic gastric distention

**Figure 4:**
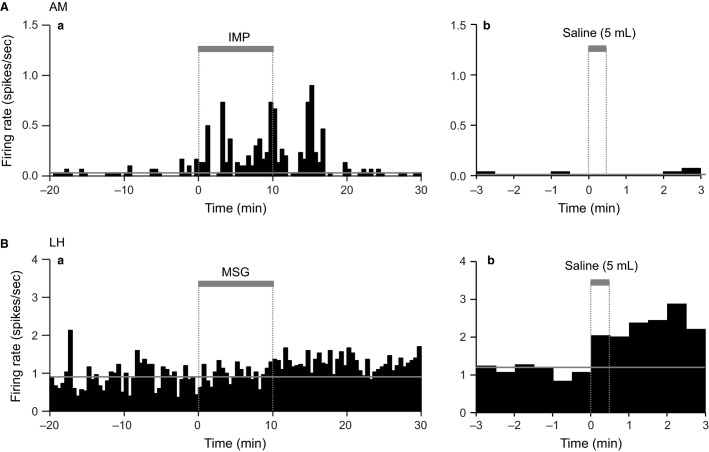
Examples of neurons that exhibited differential responses to the test solutions and phasic gastric distention in intact rats. (A) An amygdalar (AM) neuron that responded to the IMP solution but did not respond to phasic gastric distention. (B) An LH neuron that did not respond to the MSG solution, but responded to phasic gastric distention (5 mL saline flush). Thick gray bars on the histogram indicate solution administration. Horizontal thin gray lines indicate the mean baseline firing rates.

The above responses could be ascribed to stomach distention due to the intragastric administration of the test solutions. To investigate this possibility, some neurons were tested with a flush of physiological saline (5 mL) after recording the responses to the test solutions. Figure4A shows an example of a neuron that responded to the IMP solution, but not to a flush of physiological saline. This suggests that the neural responses to the IMP solution were not ascribed to phasic gastric distention. Figure4B shows an example of a neuron that did not respond to the MSG solution, but did respond to a flush of physiological saline. This suggests that the neuron was more sensitive to phasic gastric distention induced by the flush. Table3 shows the summary of the responses to phasic gastric distention. In total, the ratios of neurons that responded similarly to both the test solutions and the saline flush were 9.4% (9/96) and 12.5% (8/64) in the amygdala and LH, respectively; these data suggest that phasic gastric distention was not a major factor in the responsiveness of neurons to the test solutions.

**Table 3:** The number of neurons that responded similarly to phasic gastric distention and a given test solution in the same direction (excitatory or inhibitory) (numerator) out of the number of neurons that responded to a test solution (denominator) in intact rats

	MSG	IMP	MSG + IMP	Saline	NaCl
AM	4/18	1/27	0/12	0/14	4/25
LH	0/14	1/12	3/12	2/19	2/7

All neurons were tested with both the test solutions and phasic gastric distention.

AM, amygdala; LH, lateral hypothalamus; Saline, physiological saline.

### The effects of vagotomy on neuronal responses to MSG and NaCl solutions: response characteristics

In SVX rats, 104 and 90 neurons were recorded in the amygdala and LH, respectively. Table4 summarizes the number of responsive and nonresponsive neurons to the MSG or NaCl solutions in each of the brain regions in SVX rats. When the ratios of the responsive neurons were compared between SVX and intact rats, the ratio of nonresponsive neurons to the MSG solution was significantly lower in the amygdala of the SVX rats compared to the intact rats (residual analysis, standard residual = 2.575). In the LH, the ratios of the inhibitory responsive neurons to the MSG solution were significantly higher in the SVX rats than in the intact rats (residual analysis, standard residual = 3.027).

**Table 4:** The number of neurons of each response type (excitatory, inhibitory, and complex) in response to the MSG and NaCl solutions in SVX rats

	MSG	NaCl
AM		
Excitatory	15 (38%)	19 (30%)
Inhibitory	6 (15%)	6 (9%)
Complex	0 (0%)	2 (3%)
No response	19 (48%)*	37 (58%)
Total	40	64
LH		
Excitatory	10 (23%)	10 (22%)
Inhibitory	16 (36%)*	13 (28%)
Complex	0 (0%)	0 (0%)
No response	18 (41%)*	23 (50%)
Total	44	46

* Significant difference from the intact rats (residual analysis, standard residual > 2.0).

AM, amygdala; LH, lateral hypothalamus; Saline, physiological saline.

Table5 summarizes the mean latencies, durations, and magnitudes of the responses in the SVX rats. Three-way ANOVAs (group × brain region × solution) revealed several effects of SVX on the response parameters. First, in an analysis of the response latencies of the excitatory responsive neurons, there were significant interactions between group and solution (*F*(1, 110) = 4.725, *P *=* *0.032) and between brain region and group (*F*(1, 110) = 4.991, *P *=* *0.028). Post hoc comparisons indicated that the mean latencies in response to the NaCl solution were shorter in the SVX rats (intact: 13.7 ± 1.7 min; SVX: 9.0 ± 1.5 min; *P *=* *0.044, simple main effect test). In addition, the mean latencies were significantly shorter in the amygdala (10.2 ± 1.1 min) than in the LH (15.2 ± 2.0 min; *P *=* *0.032, simple main effect test) in the intact rats, but not in the SVX rats (amygdala: 12.5 ± 1.3 min; LH: 10.6 ± 1.7 min; *P *=* *0.387, simple main effect test). Second, in an analysis of the response magnitudes of the excitatory responsive neurons, there was a significant interaction between region and group (*F*(1, 110) = 4.219, *P *=* *0.042). Post hoc comparisons indicated that the mean response magnitudes were significantly lower in the amygdala (0.6 ± 0.5 spikes/sec) than in the LH (3.3 ± 0.7 spikes/sec) in the SVX rats (*P *=* *0.002, simple main effect test), but not in the intact rats (amygdala: 1.4 ± 0.4 spikes/sec; LH: 1.6 ± 0.7 spikes/sec; *P *=* *0.811, simple main effect test). Third, in an analysis of the response durations of the inhibitory responsive neurons, there was a significant interaction between group and solution (*F*(1, 65) = 11.17, *P *= 0.001). Post hoc comparisons revealed that the mean durations of the inhibitory responses to the MSG solution were significantly longer in the SVX rats (13.0 ± 1.4 min) than in the intact rats (8.0 ± 1.6 min; *P *=* *0.024, simple main effect test). In addition, the mean durations of the inhibitory responses to the NaCl solution were significantly shorter in the SVX rats (8.4 ± 1.5 min) than in the intact rats (13.6 ± 1.6 min; *P *=* *0.019, simple main effect test). Fourth, in an analysis of response magnitudes of the inhibitory responsive neurons, there was a significant main effect of group (*F*(1, 65) = 11.23, *P *=* *0.001) and a significant interaction between group and solution (*F*(1, 65) = 5.36, *P *=* *0.024). The significant main effect indicates that the mean magnitudes were less decreased in the SVX rats than in the intact rats (intact, −8.4 ± 1.3 spikes/sec; SVX, −2.2 ± 1.2 spikes/sec). Post hoc comparisons revealed that the mean magnitudes of the inhibitory responses to the NaCl solution were significantly less decreased in the SVX rats (−1.4 ± 1.8 spikes/sec) than in the intact rats (−12.0 ± 19.3 spikes/sec, *P *<* *0.001; simple main effect test). These results indicate that SVX significantly altered the neuronal responses to the intragastric administration of MSG and NaCl solutions. Specifically, vagotomy suppressed the response durations and magnitudes of the inhibitory responses to the NaCl solution.

**Table 5:** Basic response characteristics of the excitatory and inhibitory neurons in SVX rats

	MSG	NaCl
AM		
Excitatory		
Latency (min)	15.6 ± 2.4	9.3 ± 1.5^a^
Duration (min)	9.7 ± 1.6	9.7 ± 0.9
Magnitude (spikes/sec)^b^	0.3 ± 0.1	0.9 ± 0.3
Inhibitory		
Latency (min)	12.5 ± 2.2	17.0 ± 2.1
Duration (min)	12.8 ± 2.1^a^	7.6 ± 2.2^a^
Magnitude (spikes/sec)^a^	−2.9 ± 0.4	−1.2 ± 0.2^a^
LH		
Excitatory		
Latency (min)	12.5 ± 2.8	8.8 ± 1.6^a^
Duration (min)	7.4 ± 1.7	9.9 ± 2.0
Magnitude (spikes/sec)^b^	4.9 ± 1.6	1.8 ± 0.8
Inhibitory		
Latency (min)	10.7 ± 1.1	14.2 ± 2.1
Duration (min)	13.2 ± 1.5^a^	9.3 ± 1.6^a^
Magnitude (spikes/sec)^a^	−3.2 ± 0.7	−1.5 ± 0.4^a^

Data are expressed as mean ± SEM

a, b the value is significantly different from the intact rats (a) or from the other region (b) (simple main effect analysis, *P *<* *0.05).

AM, amygdala; LH, lateral hypothalamus; Saline, physiological saline.

### The effects of vagotomy on the neuronal responses to MSG and NaCl solutions: temporal response patterns

**Figure 5:**
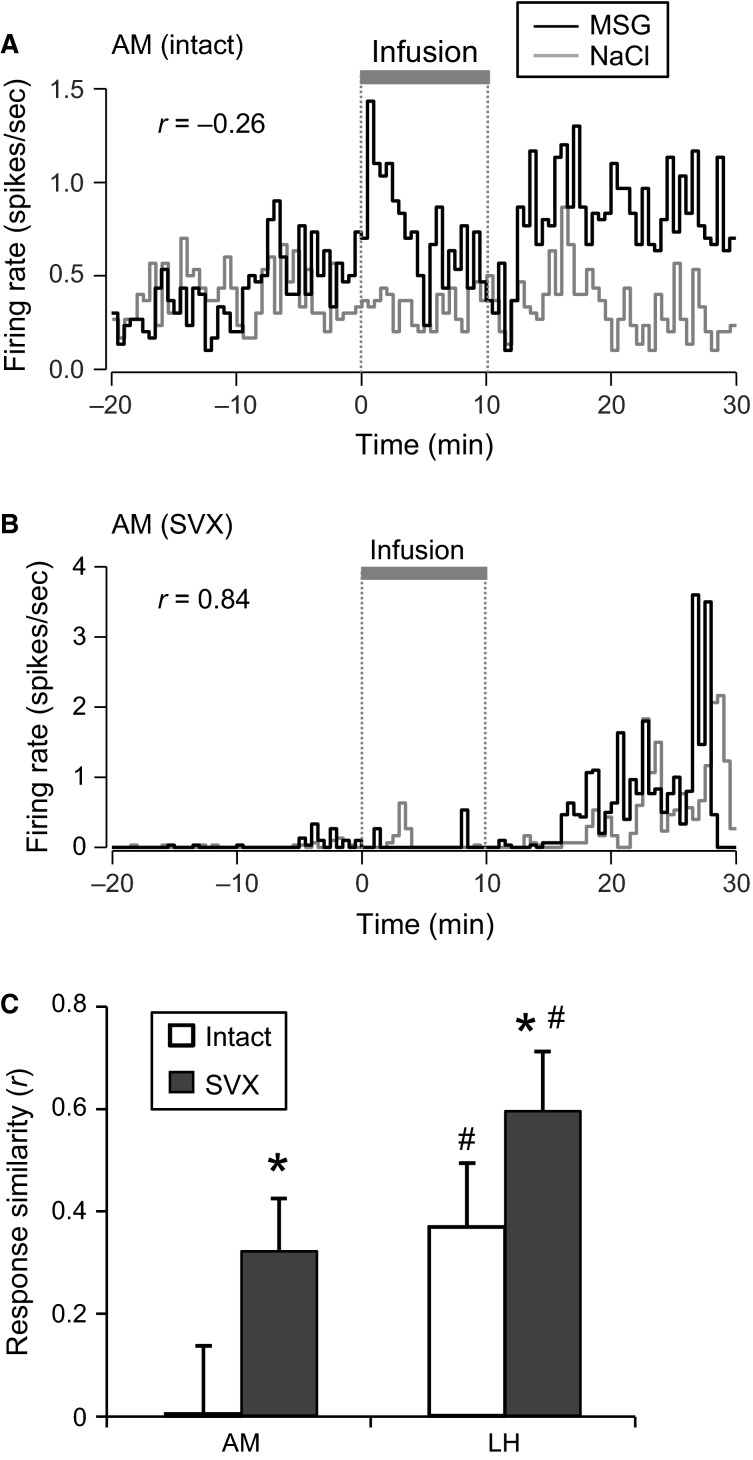
Comparison of the temporal responses patterns to MSG and NaCl solutions. Responses of amygdalar (AM) neurons recorded from an intact rat (A) and an SVX rat (B). Inset values indicate response similarities (r) between the MSG and NaCl solutions. (C) Comparison of the mean response similarities in the amygdala and LH between intact and SVX rats. Error bars represent SEM. All neurons were tested with both the MSG and NaCl solutions. *P <* *0.05, larger than intact rats in the same brain region, two-way ANOVA; ^#^*P *<* *0.05, larger than AM in the same group, two-way ANOVA.

Since some neurons were tested with both the MSG and NaCl solutions (AM in the intact rats, 11; LH in the intact rats, 11; AM in SVX rats, 14; LH in SVX rats, 6), the temporal response patterns for both solutions were compared. Figure5A shows examples of responses from an amygdalar neuron in an intact rat. The neuron exhibited excitatory responses to the MSG solution, but not to the NaCl solution. The response similarity for the two solutions using a Pearson’s correlation coefficient was *r *=* *−0.26, suggesting that the neuron responded oppositely to the two solutions. Figure5B shows examples of responses of an amygdalar neuron in an SVX rat. The neuron exhibited excitatory responses to both the MSG and NaCl solutions; the response similarity between the two solutions was *r *=* *0.84, suggesting that the neuron responded similarly to both solutions. Figure5C shows a comparison of the mean response similarities of the neurons recorded from the amygdala and LH in intact and SVX rats. A statistical comparison using a two-way ANOVA (group × brain region) revealed that there was a significant main effect of brain region (*F*(1, 39) = 6.90, *P *=* *0.018), suggesting that the neurons in the amygdala responded more differentially to the MSG and NaCl solutions. Furthermore, there was also a significant main effect of group (*F*(1, 39) = 4.40, *P *= 0.042). This indicates that SVX increased the similarity between the MSG and NaCl solutions, suggesting that SVX impaired the differential responses between the MSG and NaCl solutions. There was no significant interaction between group and brain region (*F*(1, 39) = 0.12, *P *= 0.727). The mean baseline firing rates were similarly compared using a two-way ANOVA (group × brain region). The ANOVA revealed no significant main effects (main effect of group, *F*(1, 82) = 1.65, *P *=* *0.203; main effect of brain region, *F*(1, 82) = 0.18, *P *=* *0.676), nor a significant interaction between group and brain region (*F*(1, 82) = 0.58, *P *=* *0.449).

### Recording sites of the neurons

**Figure 6:**
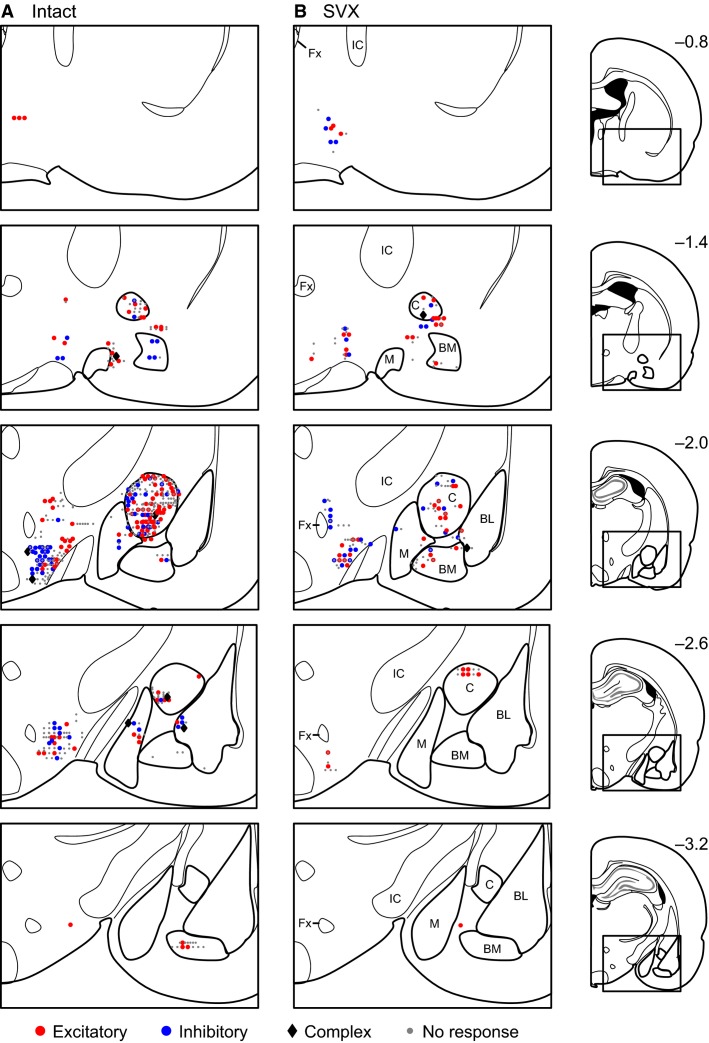
Recording locations. Each symbol represents the location of excitatory (red circle), inhibitory (blue circle), or complex (black diamond) responsive neurons or nonresponsive (gray dot) neurons recorded in an intact (A) or SVX (B) rats. Each value on the left side of each section indicates the distance (mm) from bregma. BL, basolateral nucleus of the amygdala; BM, basomedial nucleus of the amygdala; M, medial nucleus of the amygdala; C, central nucleus of the amygdala; IC, internal capsule; Fx, fornix.

Figure6 shows the recording sites of the neurons in the intact (A) and SVX (B) rats. The recorded neurons were located in the central, medial, basomedial, and basolateral nuclei of the amygdala (particularly in and around the central nucleus of the amygdala) and the LH. Neurons with each type of the responses were distributed throughout the recording regions.

## Discussion

### Responsiveness to gastric infusion of umami solutions

In the present study, similar percentages (30–60%) of neurons in the amygdala and LH responded to intragastric infusion of various umami solutions (Table1). It has been reported that the gastrointestinal tract interacts with the brain through the parasympathetic and sympathetic nervous systems, as well as various humoral factors (Mayer 2011; Critchley and Harrison 2013). Neurophysiological studies have reported that there are chemo-, osmo-, sodium-, and mechano-sensors in the gastrointestinal tract (Rogers et al. 1979; Mei 1985), while histochemical studies have reported the existence of taste-sensing receptors (T1R1/T1R3) and metabotropic glutamate receptors 1 (mGluR1) in the gastrointestinal tract of the mouse and rat, which may detect luminal glutamate (Dyer et al. 2005; Bezencon et al. 2007; San Gabriel et al. 2007). Furthermore, T1R1/T1R3 can detect both glutamate and IMP (Li et al. 2002; Nelson et al. 2002). Consistent with the presence of these receptors, afferents of the vagus nerve respond to intragastric infusion of MSG and IMP (Niijima 2000; Uneyama et al. 2006; Kitamura et al. 2011). However, approximately 90% of the amygdalar and LH neurons that responded to the test solutions did not respond to phasic gastric distention (Table3), suggesting that most neurons may respond to the chemical and osmotic factors of the stimuli rather than gastric distension. These results provide the first neurophysiological evidence that amygdalar and LH neurons respond to postingestive cues.

There were some differences in the response patterns to the test solutions between the amygdala and LH. Most amygdalar neurons exhibited excitatory responses, while the ratios of excitatory responsive neurons were similar to those of inhibitory responsive neurons in the LH. Previous neurophysiological studies have reported that most amygdalar neurons display excitatory responses to various sensory stimuli (e.g., visual, auditory, somatosensory, and taste) in rats and monkeys (Nishijo et al. 1988a,b, 1998; Uwano et al. 1995), while LH neurons exhibit similar ratios of excitatory and inhibitory neurons in rats (Fukuda et al. 1986; Ono et al. 1986). Furthermore, the response latencies of the excitatory responsive neurons were shorter in the amygdala than the LH in the present study (Table2). This suggests that the visceral information may be processed in the amygdala, and then transferred to the LH. A comparison of visual latencies reported similar results, such that response latencies to visual stimuli were longer in the LH than the amygdala, suggesting that visual information is transferred from the amygdala to the LH (Rolls 1978). These findings suggest that information from the gut is similarly processed in the amygdala and LH much like other sensory information.

The LH receives vast afferent inputs from various brain areas including the amygdala and brainstem, where various neuropeptides, acetylcholine, catecholamines as well as fast-acting neurotransmitters (i.e., GABA and glutamate) underlie synaptic transmission (van den Pol 2003; Berthoud and Münzberg 2011; Schöne and Burdakov 2012). Recent studies reported that these neuropeptidergic, cholinergic, and aminergic fibers coexpress fast-acting transmitters, and suggest that their synaptic transmission might be mediated by glutamate and GABA (van den Pol 2003; Schöne and Burdakov 2012). In the LH, both glutamatergic (excitatory) and GABAergic (inhibitory) neurotransmission is suggested to be involved in feeding behavior or aversion to food (Stanley et al. 2011; Jennings et al. 2013), and motivated behaviors might depend on balance in the activity of glutamate and GABA within the LH (Stanley et al. 2011). Therefore, an interaction between excitatory and inhibitory neurons in the LH, which receive gut information, might be important to induce motivated behaviors.

### The effects of SVX on response characteristics

SVX significantly affected the responses to the MSG and NaCl solutions. However, vagotomy could delay gastric emptying, which might affect neuronal responses in SVX rats. However, deficits in gastric emptying might gradually recover after subdiaphragmatic vagotomy (Gutierrez et al. 1971). In liquid drinking, vagotomized rats drank the same amount of water as that of sham-operated rats 14 days after vagotomy or during 125 days after vagotomy (Mordes et al. 1979; Jiang et al. 2013). This suggests that inhibition of gastric emptying by vagotomy might be small in liquid drinking (Jiang et al. 2013). In the present study, we usually recorded neurons 2–5 weeks after vagotomy. These findings suggest that changes in gastric emptying by vagotomy might not be major factors that induced differences in neuronal responses between intact and SVX rats. In addition, the recording sites differed between intact and SVX rats (Fig.6); the anterior LH neurons were more frequently sampled in SVX rats, while the central amygdalar neurons were less frequently sampled in SVX rats. The differences in the response properties between the intact and SVX rats might be ascribed to the difference in the sample sizes. To investigate this issue, we divided the neurons recorded from SVX rats into four groups according to the recording sites, as follows: anterior LH (*n *=* *13; AP < −1.2), posterior LH (*n *=* *77; AP > −1.2), CeA (*n *=* *65; neurons in the central nucleus of the amygdala), and non-CeA (*n *=* *39; neurons in the other areas of the amygdala). We then compared response properties between anterior and posterior LH or between CeA and non-CeA, and the all statistical tests were not significant or showed opposite results to those predicted from the assumption that the sampling bias affected the statistical results (data not shown). These findings suggest that it is unlikely that the observed differences were ascribed to the differences in the sample sizes in the different recording sites.

In the present study, response ratios of nonresponsive neurons to the MSG solution were decreased in SVX rats (Table4), and the response latencies, duration, and magnitudes were altered in SVX rats (Table5). Specifically, the duration of inhibitory responses to the NaCl solution were shorter, and the response magnitudes of the inhibitory responses to the NaCl solution were smaller (less negative) in the amygdala and LH of SVX rats (Table5). These data suggest that inhibitory responsive neurons received information regarding the NaCl solution from the vagus nerve. It has been reported that vagotomy can suppress water drinking in response to gastric NaCl infusion, suggesting that the vagus nerve sends information about gastric NaCl load to the brain to induce water drinking (see a review by Rowland [Rowland 2004]). These inhibitory responsive neurons may be partly involved in this phenomenon. Gastric vagal afferents, which sense dietary sodium, may inhibit a specific set of neurons (“HSD2 neurons”) involved in sodium intake in the nucleus tractus solitaries (NTS) (Shin and Loewy 2009b; Shin et al. 2009a). These results suggest that the amygdalar and LH inhibitory responsive neurons, which may receive vagal information through the NTS, could induce water drinking and inhibit sodium intake. Taken together, these findings indicate a significant contribution of the vagus nerve to amygdalar and LH neural responses for postingestive consequences. Further studies are required to confirm these hypotheses; to identify afferent inputs to the amygdala and LH from NTS, recording of amygdalar and LH neurons after NTS lesions under stimulation of the vagus nerve would be required; to investigate a role of HSD2 neurons in water drinking, recording of HSD2 neurons during infusion of MSG and NaCl solutions with concomitant measurements of fluid intake would be required.

However, vagotomy did not completely eliminate neuronal responses to the solutions. The brain receives information from the gastrointestinal tract not only through the parasympathetic (vagal) nervous system, but also through the spinal (dorsal root) afferents in the splanchnic nerve (Berthoud 2004), as well as various humoral factors (Mayer 2011; Critchley and Harrison 2013). Furthermore, both the vagus and sympathetic nerves can respond to gastric distention, luminal sodium (or osmotic stimuli), and glucose (Mei 1985; Grundy and Scratcherd 1989; Choi-Kwon and Baertschi 1991; Barone et al. 1995). These multiple routes for information processing may support the existence of neuronal responses to the test solutions after vagotomy.

### The effects of SVX on temporal response patterns

In the present study, the response similarity between the MSG and NaCl solutions was lower in the amygdala than the LH (Fig.5). It is noted that both the MSG and NaCl solutions include sodium cations and the gastrointestinal tract has receptors for glutamate (see above), suggesting that similarity between the MSG and NaCl solutions reflects postingestive effects of sodium cations, and that dissimilarity (i.e., low similarity) between the MSG and NaCl solutions reflects postingestive effects of glutamate. These findings suggest that the amygdala is more important for coding postingestive effects that are specific for glutamate. Animals can develop preferences for taste or flavor, which is paired with an intragastric infusion of nutrients in the conditioned taste/flavor preference task (Sclafani 2001). This suggests that luminal nutrients induce specific postingestive effects on the brain, including rewarding effects. Behavioral studies have reported that amygdalar lesions abolish the learning of an association between a flavor and an intragastric infusion of nutrients (Touzani and Sclafani 2005). However, rats with LH lesions could learn this association, although their preference was weaker than the sham control (Touzani and Sclafani 2002). These findings suggest that the amygdala is more critical than the LH for the acquisition of this association. Since the amygdala receives massive olfactory inputs (Swanson and Petrovicha 1998), our results suggest that the association between a flavor and the postingestive rewarding effects of glutamate is formed in the amygdala.

Furthermore, SVX increased the response similarity between NaCl and MSG solutions in the amygdala and LH (Fig.5), suggesting that the information regarding the postingestive effects of MSG is conveyed by the vagus nerve. Behavioral studies have indicated that rats develop a preference to a flavor when it is paired with an intragastric infusion of 60 mmol/L MSG, and that the acquisition of this conditioning can be disturbed by a vagotomy (Uematsu et al. 2010). In addition, fMRI studies have reported that intragastric infusion of MSG activates the forebrain, including the amygdala and LH, and that this activation was reduced by vagotomy (Tsurugizawa et al. 2008; Uematsu et al. 2010). These findings suggest that MSG-responsive neurons in the amygdala as well as the LH contribute to conditioned flavor preference learning.

### Perspectives and significance

The present results demonstrate that amygdalar and LH neurons respond to luminal MSG and NaCl, and that these response characteristics can be altered by vagotomy. These results are consistent with a pivotal role for the vagus nerve as an interface between the various sensors in the gastrointestinal tract and the brain (Berthoud and Neuhuber 2000). This visceral information is integrated by the limbic system, including the amygdala and LH, to shape emotion and control motivated feeding behavior (Mayer 2011; Critchley and Harrison 2013; Damasio and Carvalho 2013). Since intragastric infusion is rewarding enough to form conditioned flavor preference (Uematsu et al. 2009), responses to MSG in the amygdala and LH may reflect the rewarding effects of luminal MSG. Furthermore, amygdalar and LH neurons were reported to respond to an MSG solution on the tongue as well as a conditioned auditory cue associated with MSG (Nishijo et al. 1998; Tamura et al. 2000). Convergent taste and visceral information in the amygdala and LH, as well as other sensations, may contribute to the motivated behavior to ingest MSG.

## Conflict of Interest

T.K. is an employee of Ajinomoto Inc., which provided MSG and IMP. The other authors declare no conflicts of interest, financial or otherwise.
